# Glomerular Filtration Rate Estimation With Commonly Used Methods Among Healthy Live Kidney Donors of South Punjab, Pakistan

**DOI:** 10.7759/cureus.19588

**Published:** 2021-11-15

**Authors:** Suhail Iqbal Malik, Zain Ul Abideen, Muhammad Fiyaz Alam, Raheel Khan, Rashid Habib, Syed Umair Shah

**Affiliations:** 1 Department of Nephro Urology Dialysis & Renal Transplantation, Bahawal Victoria Hospital, Quaid-e-Azam Medical College, Bahawalpur, PAK; 2 Department of Nephro Urology Dialysis & Renal Transplantation, Bahawal Victoria Hospital, Quaid e Azam Medical College, Bahawalpur, PAK

**Keywords:** allograft, diethylene triamine pentaacetic acid renal scan, creatinine clearance, creatinine, glomerular filtration rate

## Abstract

Background

Accurate estimation of the donor’s glomerular filtration rate (GFR) is crucial for not only ensuring the medical appropriateness of the donor but also for the prediction of future allograft performance. The aim of this study was to compare the GFR estimation formulas and 24-hour urine creatinine clearance with diethylene triamine pentaacetic acid (DTPA) renal scan GFR.

Methods

This cross-sectional study was done at the Department of Nephro Urology Dialysis & Renal Transplantation, Bahawal Victoria Hospital, Quaid e Azam Medical College, Bahawalpur, Pakistan from September 2018 to September 2021. A total of 92 potential healthy live-related kidney donors of both genders, aged 18 to 60 years having body mass index below 35 kg/m^2^ were included. GFR was calculated with modification of diet in renal disease (MDRD), Cockcroft-Gault (CG), chronic kidney disease epidemiology (CKD-EPI) equations as well as by 24-hour urine creatinine clearance. DTPA renal scan was done to record GFR findings. GFR was compared using analysis of variance (ANOVA) among different methods.

Results

Out of a total of 92 individuals, 49 (53.3%) were male and 43 (46.7%) female. Mean age and BMI were noted to be 34.62±10.57 years and 24.40±2.71 kg/m^2^, respectively. Statistically significant differences existed between various methods of GFR estimation (p<0.001). Mean GFR as per DTPA renal scan findings was noted to be 97.32±9.39 ml/min/1.73 m^2^. Difference of 31.48±20.81, 27.37±21.1, 23.38±6.38, 15.52±37.52 was noted in estimated GFR (ml/min/1.73 m^2^) with CG formula, MDRD formula, EPI-CKD formula and 24-hour urine creatinine clearance respectively when compared with DTPA renal scan findings. The highest proportion of patients was seen with normal GFR with DTPA renal scan findings as 83 (90.2%) individuals while 24-hour urine creatinine clearance observed these to be 59 (64.1%), CG EPI-CKD formula 44 (47.8%), MDRD formula 39 (42.4%) and 40 (43.5%) with CG formula.

Conclusion

None of the GFR estimation methods resulted in similar findings. With reference to the DTPA renal scan, 24-hour urine creatinine clearance was the closest GFR estimation followed by CKD-EPI and MDRD equations.

## Introduction

Accurate estimation of the donor’s glomerular filtration rate (GFR) is crucial for not only ensuring the medical appropriateness of the donor but also for the prediction of future allograft performance. Researchers have pointed out higher GFR of the donors to be independently linked with better allograft outcomes [1]. Multiple approaches exist regarding the assessment of kidney functions while the majority of settings adopt 24-hour urinary creatinine clearance (urine-CrCl) or creatinine-based GFR estimations like “Modification of Diet in Renal Disease (MDRD)” or “Cockcroft-Gault (CG) equation” [2,3]. Insulin clearance is regarded as the “gold standard” for measuring the GFR but researchers have noted creatinine clearance to closely correlate with insulin clearance [4,5]. Insulin clearance is laborious and involves a continuous intravenous infusion, multiple repeat blood and urine collections, and careful timing of blood sampling. The radionuclide diethylene triamine pentaacetic acid (DTPA) technetium 99m scan is convenient and inexpensive and when compared to estimated GFR by serum creatinine methods, researchers have found DTPA scan-based GFR to give a better measurement of renal functions as well as better prediction of risk of mortality [6,7]. Adopting 24-hour urine collection is not always easy which can limit the reliability of this utility for measuring GFR.

In Pakistan, a study comparing the accuracy of GFR estimation formulas with 24-hour urine creatinine clearance among live kidney donors revealed that the accuracy of the MDRD formula was 49%, CG formula 42% and chronic kidney disease epidemiology (CKD-EPI) 78% [8]. The authors also concluded that 24-hour urine creatinine clearance should be utilized for evaluating GFR among live kidney donors. The DTPA scan is known to be a radio-pharmaceutical choice as it is excreted through glomerular filtration is known to give an exact measurement of GFR among adults [9]. Scarcity of literature exists comparing different GFR estimation formulas and 24-hour urine creatinine clearance with DTPA renal scan estimation of GFR so the present study was planned. The aim of this study was to compare the GFR estimation formulas and 24-hour urine creatinine clearance with DTPA renal scan GFR.

## Materials and methods

This cross-sectional study was done at the Department of Nephro Urology Dialysis & Renal Transplantation, Bahawal Victoria Hospital, Quaid e Azam Medical College, Bahawalpur, Pakistan from September 2018 to September 2021. Approval from Institutional Ethical Committee was acquired for this study as reference number QAMC/IRB/2020-416. Informed and written consent was acquired from all study participants and confidentiality of their data was ensured.

Inclusion criteria were all potential healthy live-related kidney donors of both genders, aged 18 to 60 years having body mass index below 35 kg/m^2^. All individuals having a history of primary renal or systemic diseases were excluded. Pregnant women or those who could not provide accurate timely urine collection were also not included. During the study period, a total of 92 individuals fulfilled the inclusion/exclusion criteria so they were included.

A detailed history was acquired from all study participants and a detailed physical examination was done to exclude any possible renal or related systemic illness. The height of the individuals was measured using measuring tape while weight was measured with an electronic weighing scale and BMI was calculated. A venous blood sample was taken by a well-trained phlebotomist in a vacuum tube whereas serum creatinine was measured by adopting an automated chemistry analyzer. GFR was calculated with MDRD, CG as well as the CKD-EPI formulas. All participating individuals in this study were asked to collect urine for 24 hours in a container provided by the study institution. These urine collections were sent to the institutional laboratory and GFR based on 24-hour urine creatinine clearance was noted. For the DTPA renal scan, all individuals were fully hydrated (300 to 500 ml of regular drinking water) 20 to 30 minutes prior to the renal scan. All individuals were placed in the supine position with the probe placed at the lower back and SPECT field of view involving both kidneys and the bladder. Immediately following intravenous injection of 5 mCi Tc-99m DTPA, images were taken in the front and rear positions of the dual probe. Following this, an empty syringe was collected for six seconds to obtain the residual drug count. The region of interest (ROI) was drawn manually on the frame of the kidney and the semi-lunar background was placed around the lower, outer renal margin. After the patient’s data about weight and height were input into an online computer, the GFR was automatically calculated by commercially available software according to the Gate’s algorithm.

A special proforma was designed to record all study data. Statistical Package for the Social Sciences (SPSS), version 26.0 (IBM Corp., Armonk, NY) was used for data analysis. Quantitative data like age, BMI and GFR were expressed as mean and standard deviation whereas qualitative data like gender were expressed as frequency and percentage. GFR was compared using analysis of variance (ANOVA). A P-value below or equal to 0.05 was considered statistically significant.

## Results

Out of a total of 92 individuals, 49 (53.3%) were male and 43 (46.7%) female. Mean age was noted to be 34.62±10.57 years ranging between 18 and 57 years while 55 (59.8%) patients were aged below or equal to 35 years. Mean BMI was noted to be 24.40±2.71 kg/m^2^ ranging between 18.1 and 35 while 53 (57.6%) individuals had BMI below or equal to 25. Mean serum creatinine was observed to be 0.69±0.14 ranging between 0.40 and 0.92 mg/dl. Table 1 shows the comparison of mean GFR ml/min/1.73m^2^ and it was found that statistically significant existed in between various methods of GFR estimation (P<0.001).

**Table 1:** Comparison of mean GFR (ml/min/1.73 m2) using analysis of variance (ANOVA). CG: Cockcroft-Gault equation; GFR: glomerular filtration rate; CKD-EPI: chronic kidney disease epidemiology; DTPA: diethylene triamine pentaacetic acid; MDRD: modification of diet in renal disease.

CG formula	MDRD formula	CKD-EPI formula	24-Hour urine creatinine clearance	DTPA renal scan	P-value
128.8±30.20	124.69±30.49	120.70±15.77	112.84±46.91	97.32±9.39	<0.001

**Figure 1:**
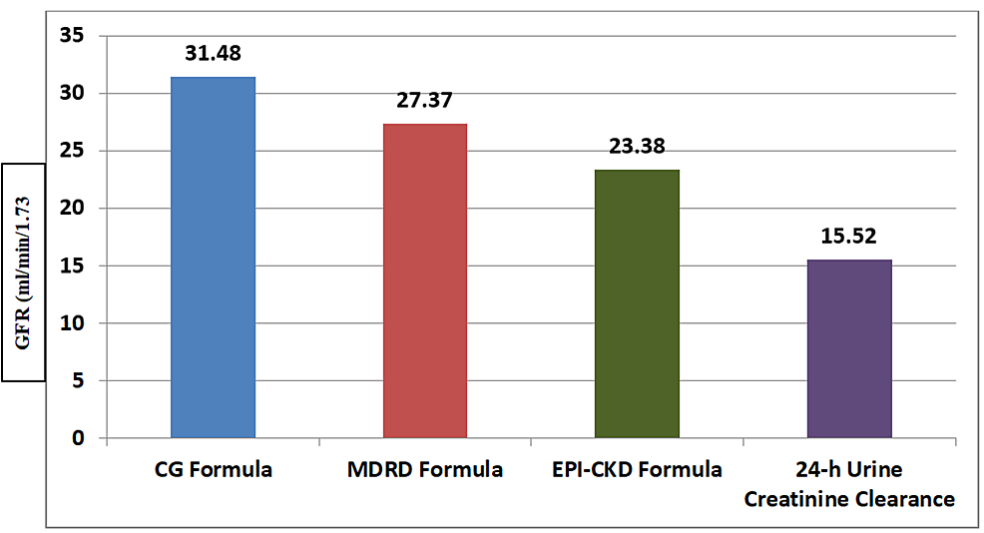
Mean difference in GFR (ml/min/1.73 m2) with various methods of GFR estimation when compared with DTPA renal scan GFR findings. CG: Cockcroft-Gault equation; GFR: glomerular filtration rate; CKD-EPI: chronic kidney disease epidemiology; DTPA: diethylene triamine pentaacetic acid; MDRD: modification of diet in renal disease.

Mean GFR as per DTPA renal scan findings was noted to be 97.32±9.39 ml/min/1.73 m^2^. Difference of 31.48±20.81, 27.37±21.1, 23.38±6.38, 15.52±37.52 was noted in estimated GFR (ml/min/1.73 m^2^) with CG formula, MDRD formula, CKD-EPI formula and 24-hour urine creatinine clearance, respectively, when compared with DTPA renal scan findings. Figure 1 shows the mean GFR difference of various methods of GFR estimation in comparison to DTPA renal scan values.

## Discussion

The classic approach to measuring GFR has been the measurement of insulin clearance which is hectic and not feasible in daily practice routines. Due to these reasons, various equations aimed to estimate GFR have been developed. The oldest well-known method of GFR calculation has been the CG equation which actually calculates creatinine clearance rather than GFR. The late 1990s saw the first description of the MDRD equation come to light, which consisted of six variables and revolutionized GFR estimation. More recently, the next advancement in GFR estimation was seen with the help of the CKD-EPI equation [10]. This study is the first one from Pakistan that compared commonly adopted methods of GFR calculation and compared these with DTPA renal scan findings in healthy adults from Pakistan.

Buchler M et al and Fotopoulos A et al in their findings shared that DTPA renal scan is the best way to get GFR estimation and has an excellent correlation with the gold standard techniques [11,12]. In the present study, we found that statistically significant existed in findings of the various methods of GFR estimation (P<0.001). Mean GFR as per DTPA renal scan findings was noted to be 97.32±9.39 ml/min/1.73 m^2^. Difference of 31.48±20.81, 27.37±21.1, 23.38±6.38, 15.52±37.52 was noted in estimated GFR (ml/min/1.73 m^2^) with CG formula, MDRD formula, CKD-EPI formula and 24-hour urine creatinine clearance, respectively, when compared with DTPA renal scan findings. As there are numerous ways to measure GFR directly, these different methods often yield measurements that do not agree with each other. For example, examination of the Bland-Altman plot of a study of nonradioactive iohexol (plasma) versus insulin (urinary) clearance suggested that the two readings often disagreed by as much as 20% [13]. Another study showed that among patients with preserved GFR, iothalamate-measured GFR was approximately 20 ml/min per 1.73 m^2^ higher than insulin-measured GFR (127.1±12.4 versus 108.3±14.1 ml/ min per 1.73 m^2^) [14]. In the literature, any direct measure of GFR is typically taken to be authentic, without much attention paid to the details of the methodology. For example, in the study by Murata et al, measured GFR was determined by using a single-period urine and plasma measurement, which may limit the accuracy of the measurement [15]. In contrast, some studies have often used multiple period measures to reduce the impact of errors in the timing of blood and urine sampling [16,17]. As per the findings of this study, the findings of the 24-hour urine creatinine clearance were the closest with a difference of 15.52±37.52 ml/min/1.73 m^2^. The 24-hour urine creatinine clearance is popularly regarded as the most adopted method for estimation of GFR and some researchers have compared it with other formula equations for the estimation of GFR establishing the authenticity of 24-hour urine creatinine clearance [8]. But, we found that 24-hour urine creatinine clearance revealed only 64.1% of patients with normal GFR findings in comparison to 90.2% with DTPA renal scan. Perrone et al revealed a lack of accuracy by 24-hour creatinine clearance varying dramatically with the true GFR measured by renal scintigraphy [18]. The most important factor with variation in findings with 24-hour urine creatinine clearance among healthy live kidney donors could be the inappropriate urine collection. Contrary to our findings, Xie P et al from China shared that DTPA renal dynamic imaging findings were found to be less accurate than the CKD-EPI equation among patients of chronic kidney disease [19]. The reasons could have been different sets of patients as they considered chronic kidney disease patients while differences in laboratory estimation parameters and different demographical findings could have been other reasons.

Star et al shared that serum creatinine concentration is influenced by muscle mass, dietary protein intake, and gender and age variations, and these factors can have limiting effects on the true estimation of GFR adopting creatinine-based approaches [20]. Among individuals with reduced GFR, it is believed that tubular secretions of creatinine rise that in turn can result in overestimation of the GFR through methods like CG equation [21,22]. A local study concluded the MDRD equation to give a better estimation of GFR when compared to the CG equation [23].

One of the limitations of this study was that being a single-center study with comparatively small sample size, our findings cannot be generalized. We were unable to take note of renal volumes among study individuals.

## Conclusions

None of the GFR estimation methods resulted in similar findings. With reference to DTPA renal scan, 24-hour urine creatinine clearance was the closest GFR estimation followed by CKD-EPI and MDRD equations. It is important to check the validity of various methods of GFR estimation among different sets of populations. Further studies should be planned to evaluate the validity of commonly adopted methods for the estimation of GFR in live kidney donors.
